# Isolation, Identification, and Growth Promotion Effects of Plant Growth-Promoting Rhizobacteria on Alfalfa

**DOI:** 10.3390/microorganisms14061275

**Published:** 2026-06-05

**Authors:** Aolei He, Bingpeng Shen, Yang Yang, Ting Wang, Ying Zhang, Ailin Li

**Affiliations:** Grassland Ecosystem Key Laboratory of Ministry of Education, College of Pratacultural Science, Gansu Agricultural University, Lanzhou 730070, China; 1073325020079@st.gsau.edu.cn (B.S.); yang_yang_yaf@163.com (Y.Y.); wangting921221@163.com (T.W.); 1073323120125@st.gsau.edu.cn (Y.Z.); 1073325120262@st.gsau.edu.cn (A.L.)

**Keywords:** plant growth-promoting rhizobacteria, growth-promoting characteristics, microbial consortia, alfalfa

## Abstract

In this study, nine strains of plant growth-promoting rhizobacteria (PGPR) with multiple growth-promoting functions were isolated and screened from the rhizosphere of plants (*Phragmites communis*, *Triglochin maritimum*, and *Alhagi maurorum*) in the arid and barren regions of Western China. These strains belong to five genera: *Klebsiella*, *Bacillus*, *Serratia*, *Pseudomonas*, and *Flavobacterium*. The growth-promoting characteristics of these nine strains (PAP4, PA35, AC12, ACP1, AC25, TP7, TP8, TP12, and TP14) were analyzed. Furthermore, the growth-promoting potential of these PGPR strains was comprehensively evaluated through plate and pot experiments using *Arabidopsis thaliana* and alfalfa. The results indicate that most strains possess the ability to fix nitrogen and secrete zeatin and extracellular polysaccharides (EPS). Some strains exhibited significant traits such as phosphate solubilization, siderophore secretion, and the production of 1-aminocyclopropane-1-carboxylate (ACC) deaminase and indole-3-acetic acid (IAA). All strains showed high salt tolerance (0–8% NaCl) and were induced to secrete more EPS under salt stress. Plate experiments demonstrated that volatile organic compounds (VOCs) from the nine strains significantly promoted the root development of *Arabidopsis thaliana* and optimized its root architecture. Pot experiments revealed that inoculation with single strains influenced the growth of alfalfa to varying degrees; among them, strain TP14 showed the best performance, increasing plant height and shoot dry weight by 44.7% and 51.2%, respectively. Regarding microbial consortia, the combinations BD (PAP4 + TP14), ABC (PA35 + PAP4 + AC25), and ABCD (PA35 + PAP4 + AC25 + TP14) significantly improved the biomass, plant height, and stem diameter of alfalfa. The superior strains and their combinations identified in this study effectively promote plant growth. These high-performing PGPR strains provide valuable microbial resources for the development of bio-fertilizers tailored for saline–alkali and barren regions in Western China.

## 1. Introduction

Plants have established intimate interactions with indigenous soil microorganisms during long-term adaptation to their habitats [1]. The rhizosphere represents a critical zone where plants engage in signaling and material exchange with microorganisms [2]. Plant Growth-Promoting Rhizobacteria (PGPR) refers to a collective group of beneficial bacteria that can colonize the plant rhizosphere system and significantly promote plant growth or inhibit the activities of pathogenic microorganisms [3]. An increasing number of studies have shown that PGPR plays a crucial role in plant health and adaptation to various environmental challenges [4]. Therefore, mining excellent microbial resources from different habitats holds significant importance.

PGPR possess diverse plant protection mechanisms, including the synthesis of osmoprotectants (such as exopolysaccharides), production of volatile compounds and siderophores, 1-aminocyclopropane-1-carboxylate (ACC) deaminase activity, nitrogenase activity, phosphate solubilization, and phytohormone production, which directly or indirectly promote plant growth and development [5,6,7,8]. In addition to symbiotic nitrogen-fixing microorganisms (such as rhizobia), there exist various non-symbiotic nitrogen-fixing bacteria, including associative nitrogen fixers such as *Bacillus*, *Pseudomonas*, *Klebsiella*, and *Enterobacter* [9,10]. Inoculation with these nitrogen-fixing bacteria can promote the growth of various plants [11]. Studies have shown that the generally higher nitrogen use efficiency of *indica* rice varieties compared to *japonica* varieties is closely associated with the enrichment of more nitrogen metabolism-related microbial communities in the rhizosphere of the former, indicating that nitrogen-fixing microorganisms play important roles in promoting plant growth and nitrogen uptake [12]. In phosphorus-deficient environments, phosphate-solubilizing bacteria facilitate the solubilization of insoluble phosphates, which enhances plant phosphorus uptake and subsequently stimulates growth [13]. By synthesizing hormones and hormone-like substances with enzymatic properties, PGPR modulate phytohormone levels within the plant tissues, phyllosphere, and rhizosphere [14]. These microbially derived hormones exert a profound influence on plant physiological processes and facilitate effective host colonization [6,14]. Volatile organic compounds (VOCs), acting as long-distance signaling molecules that exert growth-promoting functions through non-physical contact between PGPR and plants, play an important role in helping plants alleviate biotic and abiotic stresses [7,15]. In agricultural production, PGPR can function as biofertilizers or biopesticides to create a favorable rhizosphere ecological environment for normal plant growth. While ensuring the sustainable development of modern agriculture, they also achieve the goal of increasing yields, demonstrating significant research prospects and application potential [10]. However, significant differences exist between the effects of single strains and microbial consortia in PGPR application research. Due to their limited functionality, single strains often struggle to perform effectively in complex environments and exhibit weak environmental adaptability and stability [16]. In contrast, microbial consortia, through the synergistic interactions of different functional strains, can achieve complementary growth-promoting functions, enhancing both adaptability to complex environments and the overall growth-promoting effects on plants [13]. For instance, when nitrogen-fixing, phosphate-solubilizing, and auxin-secreting bacteria are combined, the strains form a functional complementarity; their effectiveness in promoting plant growth and improving soil fertility is significantly superior to that of single strains [17]. Therefore, screening for high-efficiency growth-promoting strains from various environments and constructing functionally complementary microbial consortia is an effective way to enhance the application efficacy of microbial inoculants and promote plant growth.

Alfalfa (*Medicago sativa* L.) is known as the “Queen of Forages” due to its high protein content and excellent palatability [18]. Its symbiotic nitrogen fixation with rhizobia plays a crucial role in sustainable agricultural development; however, this process is susceptible to complex environmental factors that can limit nitrogen fixation efficiency. PGPR can enhance the nitrogen-fixing performance of rhizobia [19]. Moreover, the synergistic interaction between PGPR and rhizobia in leguminous plants can help plants withstand adverse environmental conditions. Therefore, screening PGPR strains that promote the growth of alfalfa is of particular significance. In Western China, vegetation must contend with a complex array of environmental stressors, including extreme heat, drought, salinity, and soil infertility [20,21]. The microbial wealth harbored within the rhizospheres of these plants has evolved unique symbiotic interactions, offering immense potential for practical application. Against this backdrop, this study sampled rhizosphere soil from *Phragmites australis*, *Triglochin palustris*, and *Alhagi camelorum* in Western China to isolate PGPR exhibiting diverse plant growth-promoting traits. Following taxonomic identification using 16S rRNA gene sequencing and characterization of their plant growth-promoting properties, elite individual strains and their corresponding consortia were evaluated through both in vitro plate assays and in vivo pot experiments. By determining the impact of high-efficiency strains and their optimal combinations on alfalfa growth, this research aims to provide high-quality microbial germplasm for the formulation of functional biofertilizers.

## 2. Materials and Methods

### 2.1. Experimental Material

#### 2.1.1. Soil and Plant Materials

Rhizosphere soil (the soil remaining adhered to the root system after shaking off large clumps) was collected from *Phragmites australis* (Cav.) Trin. ex Steud, *Triglochin palustris* L., and *Alhagi camelorum* Fisch. growing in Minqin County, Wuwei City, Gansu Province, China (38°03′–39°27′ N, 102°48′–104°12′ E). The samples were passed through a 200-mesh sieve and stored for subsequent use.

*Arabidopsis thaliana* seeds (maintained in our laboratory) and alfalfa (*Medicago sativa* L. cv. “Zhongmu No. 1”, purchased from Beijing Best Grass Industry Co., Ltd. Beijing, China) were used. Arabidopsis seeds were surface-sterilized with 75% ethanol and 1% sodium hypochlorite (NaClO) for 3 min each, with continuous agitation. Subsequently, the seeds were rinsed 8–12 times with sterile distilled water and stratified at 4 °C for 3 days prior to use. Alfalfa seeds were soaked in 6% NaClO for 3 min and rinsed at least 10 times with sterile water until the residual NaClO was completely removed.

#### 2.1.2. Culture Media

The media used in this study included R2A (Reasoner’s 2A Agar), LB (Luria–Bertani medium), NFM (Nitrogen-Free Medium) [17], NBRIP (National Botanical Research Institute’s Phosphate), King’s B (King’s Medium B) [22], CAS (Chrome Azurol S Agar), DF (Dworkin and Foster Minimal Salt Medium), and ADF (1-Aminocyclopropane-1-Carboxylic Acid Dworkin–Foster Medium) [23]. The compositions of these media are shown in Table 1.

### 2.2. Experimental Methods

#### 2.2.1. Isolation and Purification of Bacterial Strains

The collected rhizosphere soil was sieved inside a clean bench. A 10 g soil sample was weighed and transferred into a conical flask containing 90 mL of sterile physiological saline. The flask was incubated in a shaker at 150 r/min and 28 °C for 30 min, then allowed to stand until phase separation occurred. One milliliter of the supernatant was taken as the stock solution. Subsequently, 100 μL of the stock solution was added to 900 μL of physiological saline and serially diluted to concentrations of 10^−2^, 10^−3^, 10^−4^, 10^−5^, 10^−6^ and 10^−7^. For each dilution, 200 μL was spread onto R2A, LB, NBRIP, and NFM solid agar plates until evenly distributed; three replicates were performed for each concentration gradient. The plates were then inverted and incubated in a constant-temperature incubator at 28 °C. After 3 days, individual colonies were numbered and picked using sterile toothpicks for further cultivation. Bacterial cultures grown to the logarithmic phase were preserved in cryovials with 20% glycerol and stored at −80 °C for future use.

#### 2.2.2. Strain Identification

Bacterial cultures stored at −80 °C were purified until individual colonies appeared. The bacterial broth was then centrifuged in 1.5 mL microcentrifuge tubes; the supernatant was discarded, and the cell pellets were collected. Genomic DNA was extracted from each strain using a bacterial genomic DNA extraction kit (Omega Bio-tek, Norcross, GA, USA). Using the extracted DNA as a template, 16S rRNA gene identification was performed using the universal primers 27F/1492R. The 20 μL PCR amplification system consisted of 10 μL Taq polymerase, 0.5 μL each of the 27F and 1492R primers, 1 μL DNA template, and 8 μL deionized water. The PCR amplification conditions were as follows: an initial denaturation at 94 °C for 3 min, followed by 30 cycles of denaturation at 94 °C for 30 s, annealing at 55 °C for 30 s, and extension at 72 °C for 90 s, with a final extension at 72 °C for 10 min [6].

Sequencing of the 16S rRNA gene was conducted by Beijing Tsingke Biotech Co., Ltd., Beijing, China. The resulting sequences were submitted to the EzBioCloud database (https://www.ezbiocloud.net/identify, accessed on 23 August 2026) for homology alignment. The alignment results are summarized in Table 2.

#### 2.2.3. Detection of Plant Growth-Promoting (PGP) Traits

##### Screening of Nitrogen Fixation, Phosphate Solubilization, and ACC Deaminase Activity

The isolated strains were cultured on solid media to evaluate their nitrogen fixation, phosphate solubilization, ACC deaminase activity, and siderophore production [17]. For each strain, three spots were inoculated per plate, with three replicate plates per treatment. Strains exhibiting nitrogen fixation, phosphate solubilization, and ACC deaminase activity were further screened, resulting in a preliminary selection of nine strains. The screening assays were evaluated qualitatively based on bacterial growth and the intensity of the characteristic color reaction on the selective media. “−” indicates no detectable activity, “+” indicates a positive reaction, and “++” indicates a strong positive reaction.

##### Determination of Siderophore Production

Siderophore production was quantified following the method of Sasirekha et al. [24] with slight modifications. Bacterial cultures in the logarithmic growth phase were adjusted to an optical density of OD_600_ = 0.8. Subsequently, a 1% (*v*/*v*) inoculum was added to 30 mL of liquid mineral salt medium and incubated at 28 °C and 180 r/min for 2–3 days. A 5 mL aliquot of the culture was centrifuged at 12,000 r/min for 2 min. The supernatant was collected and mixed with an equal volume of CAS assay solution, then allowed to react in the dark at room temperature for 60–90 min. The absorbance at OD_630_ was measured in triplicate. The siderophore content was calculated as follows: siderophore Content (%) = [(Ar − A) ÷ Ar] × 100%. Where Ar is the absorbance of the blank control and A is the absorbance of the sample.

Preparation of CAS Assay Solution. Solution A: 0.079 g of Chrome Azurol S (CAS) was dissolved in 50 mL of deionized water, followed by the addition of 10 mL of 1 mmol/L FeCl_3_ solution (prepared in HCl). Solution B: 0.069 g of hexadecyltrimethylammonium bromide (HDTMA) was dissolved in 40 mL of deionized water. Solution A was slowly added to solution B along the beaker wall and stirred thoroughly to obtain 100 mL of the blue CAS assay solution.

##### Extraction of Exopolysaccharides (EPS)

Detection of exopolysaccharide production: The exopolysaccharides (EPS) of the preliminarily screened strains were extracted from the fermentation broth using the low-temperature ethanol precipitation method [25]. Strains were inoculated into a basal sugar-producing medium at a 3% (*v*/*v*) inoculum and incubated with shaking at 28 °C and 180 r/min for 5 days. Following incubation, the cultures were centrifuged at 10,000 r/min and 4 °C for 15 min; the cell pellets were discarded and the supernatants were collected. Trichloroacetic acid (TCA) was added to the supernatants to a final concentration of 10% (*w*/*v*), and the mixture was allowed to stand at a low temperature for 12 h. To remove proteins, the samples were centrifuged again at 10,000 r/min and 4 °C for 15 min. Three volumes of pre-cooled 95% ethanol were added to the resulting supernatants and mixed thoroughly. After standing at 4 °C for 24 h, the mixture was centrifuged at 10,000 r/min and 4 °C for 15 min to obtain the crude EPS precipitate.

##### Quantification of EPS Content

Quantification of EPS content: The EPS content in the fermentation broth was determined using the phenol-sulfuric acid method [26]. One milliliter of the appropriately diluted crude polysaccharide solution was mixed with 1 mL of 6% (*v*/*v*) phenol solution. Subsequently, 5 mL of concentrated sulfuric acid was added slowly and shaken thoroughly. After the mixture cooled to room temperature, the absorbance was measured at a wavelength of 490 nm [27]. A standard curve was generated using glucose as a reference to calculate the EPS concentration in the culture broth. The obtained values were used for comparative evaluation of EPS production among bacterial isolates and under different salinity treatments. To evaluate the effect of salinity on EPS production, strains were inoculated into basal sugar-producing media containing 0%, 2%, 4%, 6%, and 8% NaCl and incubated with shaking at 28 °C and 180 r/min for 5 days. The EPS content for each treatment was measured in triplicate using the aforementioned phenol-sulfuric acid method.

##### Determination of Extracellular Phytohormones

Determination of extracellular phytohormones content: The capacity of the strains to secrete auxin (IAA) and zeatin was determined using high-performance liquid chromatography (HPLC) [27]. The strains were inoculated into King’s B medium and incubated in a shaker at 28 °C and 180 r/min for 7 days, with uninoculated medium serving as the control; each treatment was performed in triplicate. The samples were extracted with ethyl acetate and concentrated to dryness using a vacuum centrifugal concentrator (RVC, Beijing, China). The residues were redissolved in 2 mL of methanol, filtered through a 0.45 μm organic phase filter, transferred into amber autosampler vials, and stored at 4 °C prior to HPLC analysis.

The HPLC chromatographic conditions were as follows: an Agilent ZORBAX Eclipse Plus C18 column (4.6 mm × 250 mm, 5 μm) was employed; the mobile phase consisted of methanol (Phase A) and a 0.2% (*v*/*v*) aqueous glacial acetic acid solution (Phase B); the column temperature was maintained at 30 °C; the flow rate was set to 0.8 mL/min; and the injection volume was 10 μL.

#### 2.2.4. Growth Promotion Experiment and Index Measurement

Arabidopsis seeds (8–10 per plate) were sown onto 90 mm Petri dishes containing 1/2 MS medium (0.2215% MS, 1% sucrose, 0.75% agar, pH 5.7) [7]. Fresh bacterial suspension (20 μL) was inoculated at a distance of 5 cm from the seeds. The plates were placed vertically on racks and maintained in a growth chamber under long-day conditions (16 h light/8 h dark), with temperatures ranging from 22 °C to 24 °C and a relative humidity of 60%.

Plump seeds of alfalfa were selected and sown in pots (12 cm diameter, 25 cm height) filled with sterilized vermiculite. Fifteen seeds were sown per pot and covered with a layer of vermiculite to ensure uniform seedling emergence. Once all seedlings had emerged, they were thinned to eight uniform plants per pot. After two weeks of growth, the seedlings were inoculated. For each treatment, 10 mL of fresh bacterial suspension or culture medium (LB) was applied to the stems using a rotary spray/wash method. Mixed inoculants were prepared in equal proportions (1:1 or 1:1:1, OD_600_ = 0.8). Based on the individual effects of the strains on the growth of Arabidopsis thaliana and Medicago sativa, as well as the relationships between their growth-promoting characteristics, four strains (PA35, PAP4, AC25, and TP14) were selected for the formulation of microbial consortia, taking the diversity of composite inoculants into account. These strains were combined into 2-strain, 3-strain, and 4-strain mixtures. The pot experiments were conducted in a growth chamber at Gansu Agricultural University under the following conditions: a 16 h/day photoperiod, light intensity of approximately 800 μmol/m^2^·s, relative humidity of 60%, and a temperature cycle of 28 ± 2 °C (day)/23 ± 2 °C (night). The plants were irrigated with Hoagland’s nutrient solution [6] every two days. Relevant physiological indices were measured after 40 days of growth.

After 40 days of growth, samples of the pot-cultured alfalfa seedlings were collected to measure relevant physiological indices. The shoots and roots were separated using scissors, and the root were thoroughly rinsed with distilled water and blotted dry with filter paper. The fresh weights of the shoots and roots were measured and recorded using an analytical balance. Subsequently, the plant materials were placed in paper envelopes and dried in an oven at 65 °C until a constant weight was reached to determine the corresponding dry weights. After removing and cleaning the plant material from each treatment, the plant height and root length of the seedlings were measured using a ruler. Stem diameter was determined using a digital vernier caliper.

Chlorophyll and carotenoid (Car) contents were determined according to the method described by Chen et al. (2018) [28]. Briefly, 0.2 g of fresh leaves were added to 10 mL of 95% ethanol to extract pigments under dark conditions at 4 °C. The absorbance values were subsequently measured at 663, 646, and 470 nm, respectively.

The root systems of Arabidopsis thaliana were scanned using a root scanning device (Perfection V700 Photo, Seiko Epson Corp., Suwa, Japan), and the resulting images were analyzed for each treatment using WinRHIZO software (version 2013e, Regent Instruments Inc., Québec, QC, Canada). During the scanning process, the roots were gently spread apart with forceps to avoid overlap between lateral roots and minimize measurement errors.

Root activity was determined using the classic Triphenyl Tetrazolium Chloride (TTC) reduction method, following the protocol described by He et al. [5]. The roots were removed from the pots and thoroughly cleaned. Root tips (0.5 g) were placed into 10 mL test tubes. A 10 mL mixture of 0.4% TTC solution and phosphate buffer (1:1, *v*/*v*) was added to the tubes, ensuring the roots were completely submerged. After incubation in the dark at 37 °C for 3 h, 2 mL of concentrated sulfuric acid (H_2_SO_4_) was added to terminate the reaction. For the blank control, the acid was added before the introduction of the root samples. The roots were then removed, blotted dry with filter paper, and transferred to test tubes containing 10 mL of methanol. The tubes were sealed and kept in the dark for 3 h until the roots were completely decolorized. The absorbance was measured at 485 nm using a spectrophotometer, with the blank test serving as the reference. The amount of TTC reduction was determined using a standard curve [29].

### 2.3. Statistical Analysis

All data in this experiment were subjected to one-way analysis of variance (ANOVA) using SPSS 19. Means and standard errors (SE) were calculated, and the significance of differences was analyzed using Duncan’s multiple range test. Data visualization and graphing were performed using Excel and Origin 9.

## 3. Results

### 3.1. Identification and Growth-Promoting Characteristics of PGPR

In this study, 29 strains of culturable bacteria were isolated from the rhizosphere soil of *Phragmites australis*, *Triglochin maritima*, and *Alhagi maurorum* using culturable bacterial isolation methods. Based on plate assays for nitrogen fixation, phosphate solubilization, auxin (IAA) secretion, and siderophore production, as well as interaction assays with *Arabidopsis thaliana*, nine strains were identified as potential candidates for high-quality microbial fertilizers. These strains are PAP4, PA35, AC12, ACP1, AC25, TP7, TP8, TP12, and TP14. Further analysis of 16S rRNA sequences revealed that these nine strains belong to the genera *Klebsiella*, *Bacillus*, *Serratia*, *Pseudomonas*, and *Flavobacterium*. Detailed information is presented in Table 3.

**Figure 1:**
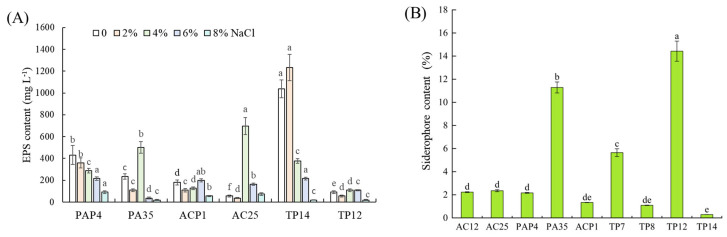
Extracellular polysaccharide (EPS) content (**A**) secreted by PGPR strains under different salt concentrations; Siderophore levels (**B**) produced by PGPR. Values are means and bars indicate standard errors (SEs) (*n* = 5). Columns with different letters indicate significant differences among treatments at *p* < 0.05 (ANOVA and Duncan’s multiple comparison test).

Initial screening of growth-promoting characteristics on solid media (Supplementary Figure S1) revealed that most strains possessed nitrogen-fixation capacity. On inorganic phosphate solubilization plates, strain TP8 exhibited the largest phosphate-solubilizing halo. Strains TP7, TP14, PA35, and ACP1 displayed smaller halos around their colonies, indicating relatively weak phosphate-solubilizing abilities, while no halos were observed around strains AC12, PAP4, AC25, and TP12. Qualitative detection of ACC deaminase showed that only strains PAP4, AC12, AC25, and TP14 were capable of utilizing ACC (Table 4). Regarding siderophore production, strain TP14 showed significant activity, whereas the color changes around the colonies of the remaining strains were weak, and the change for strain AC25 was negligible. Further quantitative analysis of siderophore content in these nine strains showed that strain TP14 had the highest content at 12.63%, while TP12 had the lowest at 0.29% (Figure 1B).

The hormone levels of these strains were determined using HPLC. Strains AC25 and TP12 did not produce zeatin, whereas the zeatin content secreted by the remaining strains ranged from 12.88 to 23.30 μg/mL (Table 4). Among them, strain AC12 exhibited the highest secretion, followed by TP14. In addition to zeatin, strain TP14 was found to secrete indole-3-acetic acid (IAA) at a concentration of 14.46 μg/mL, while no IAA production was detected in the other strains (Table 4). Evaluation of exopolysaccharide (EPS) secretion across different salinity levels indicated that strains PAP4, PA35, ACP1, AC25, TP14, and TP12 possessed strong salt tolerance, maintaining growth at NaCl concentrations ranging from 0% to 8%. High salinity did not inhibit the survival of these strains, although their growth rates decreased. Quantitative analysis of EPS content showed that for strains PA35 and AC25, a 4% NaCl concentration stimulated increased EPS secretion. In contrast, strain TP14 reached its peak EPS production of 1233.93 mg/L at 2% NaCl. Although TP14 grew well at other salt concentrations, its EPS secretion decreased compared to the control (Figure 1A). These results suggest that these three strains possess robust self-protection mechanisms under salt stress.

### 3.2. Effects of PGPR Volatile Organic Compounds (VOCs) on the Growth of Arabidopsis thaliana

**Figure 2:**
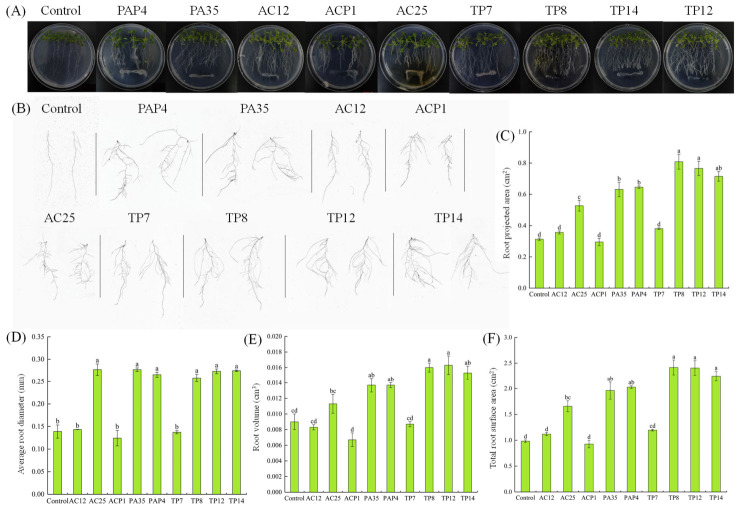
Effects of PGPR-derived volatile organic compounds (VOCs) on the root system development of *Arabidopsis thaliana*. Growth phenotypes of *Arabidopsis thaliana* (**A**); Scanned images of the root system (**B**); Root projected area (**C**); Average root diameter (**D**); Root volume (**E**); Total root surface area (**F**). Values are means and bars indicate standard errors (SEs) (*n* = 6). Columns with different letters indicate significant differences among treatments at *p* < 0.05 (ANOVA and Duncan’s multiple comparison test).

Plate assays were conducted to further verify the effects of VOCs from the aforementioned strains on plant growth. As shown in Figure 2A,B, the nine strains significantly promoted the growth of Arabidopsis seedlings, with a particularly pronounced effect on root system development. According to root scanning and WinRHIZO software analysis, the total root projected area of seedlings treated with strains AC12, AC25, PA35, PAP4, TP7, TP8, TP12, and TP14 was significantly higher than that of the control by 13.7%, 68.4%, 101.6%, 106.4%, 21.7%, 158.5%, 144.7%, and 128.4%, respectively (*p* < 0.05) (Figure 2C), with strain TP8 exhibiting the most substantial promotional effect.

The average root diameter for treatments AC12, AC25, PA35, PAP4, TP8, TP12, and TP14 was significantly higher than the control by 2.88%, 99.3%, 99.3%, 91.4%, 85.6%, 96.4%, and 97.8%, respectively (*p* < 0.05) (Figure 2D), with AC25 and PA35 showing the best performance. Root volume for AC25, PA35, PAP4, TP8, TP12, and TP14 was significantly higher than the control by 22.2%, 55.6%, 55.6%, 77.8%, 77.8%, and 66.7%, respectively (*p* < 0.05) (Figure 2E). Additionally, the total root surface area for AC12, AC25, PA35, PAP4, TP7, TP8, TP12, and TP14 significantly exceeded the control by 13.8%, 68.5%, 95.9%, 106.3%, 21.7%, 145.8%, 144.8%, and 128.6%, respectively (*p* < 0.05) (Figure 2F).

### 3.3. Effects of Inoculating Individual Strains and Their Consortia on the Growth of Alfalfa

**Figure 3:**
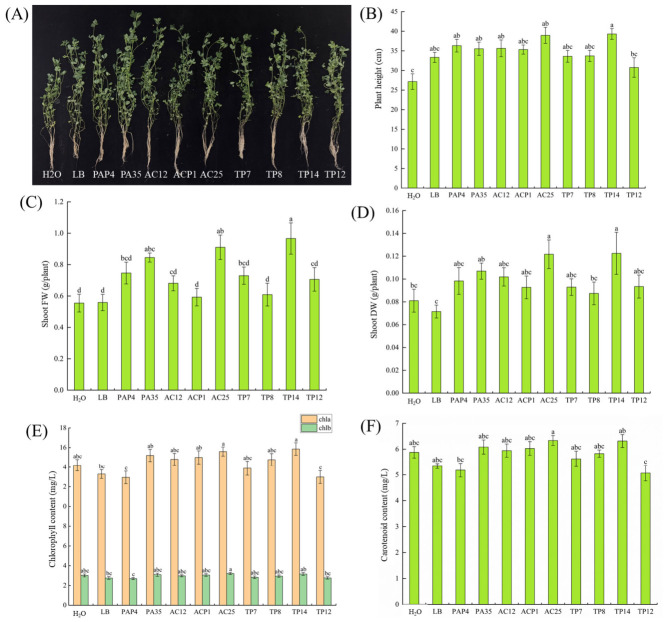
Effects of PGPR single inoculation on the growth of alfalfa (**A**). Plant height (**B**); Shoot fresh weight (**C**); Shoot dry weight (**D**); Chlorophyll content (**E**); Carotenoid content (**F**). Values are means and bars indicate standard errors (SEs) (*n* = 12). Columns with different letters indicate significant differences among treatments at *p* < 0.05 (ANOVA and Duncan’s multiple comparison test).

The effects of the nine strains on the growth of alfalfa are shown in Figure 3A. Compared to the control, inoculation with these plant growth-promoting bacteria (PGPB) promoted seedling plant height, fresh weight, and dry weight to varying degrees, with increases ranging from 13.3–44.7%, 6.8–74.1%, and 7.9–51.2%, respectively (*p* < 0.05) (Figure 3). Specifically, the plant heights for treatments PAP4, PA35, AC12, ACP1, AC25, TP7, TP8, TP14, and TP12 were higher than the control (water-inoculated treatment) by 33.7%, 30.8%, 31.2%, 30.0%, 43.4%, 23.7%, 24.1%, 44.7%, and 13.2%, respectively (*p* < 0.05) (Figure 3B).

The shoot fresh weights of the PAP4, PA35, AC12, AC25, TP8, TP14, and TP12 treatments were significantly higher than the control by 34.5%, 52.3%, 22.7%, 64.1%, 31.5%, 74.1%, and 27.2%, respectively (*p* < 0.05) (Figure 3C). Correspondingly, their dry weights exceeded the control by 21.3%, 31.9%, 25.8%, 50.2%, 14.7%, 51.2%, and 15.3% (*p* < 0.05) (Figure 3D). Furthermore, these strains influenced chlorophyll content, as illustrated in Figure 3E,F. The chlorophyll *a* content in the PA35, AC12, ACP1, AC25, TP8, and TP14 treatments was significantly higher than the control by 14.4%, 11.4%, 12.8%, 17.3%, 11.1%, and 19.3%, respectively (*p* < 0.05) (Figure 3E). Carotenoid content significantly exceeded the control by 14.1%, 11.5%, 13.1%, 18.9%, 5.49%, 9.31%, and 18.5% in the respective treatments (*p* < 0.05) (Figure 3F). Additionally, the chlorophyll *b* content of PA35, ACP1, AC25, and TP14 was 2.68%, 1.59%, 6.53%, and 5.17% higher than the control (*p* < 0.05) (Figure 3E). These results indicate that inoculating these strains into sterilized soil effectively promotes the growth of alfalfa.

**Figure 4:**
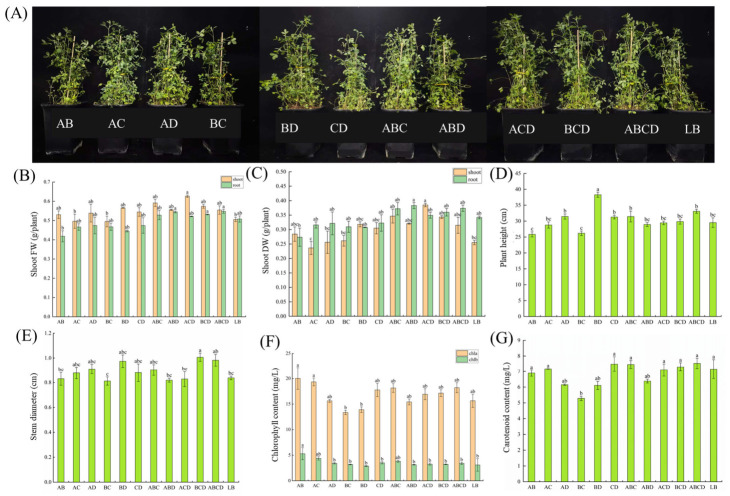
Effects of PGPR microbial inoculants on the growth of alfalfa (**A**). Shoot fresh weight (**B**); Shoot dry weight (**C**); Plant height (**D**); Stem diameter (**E**); Chlorophyll content (**F**); Carotenoid content (**G**). Values are means and bars indicate standard errors (SEs) (*n* = 12). Columns with different letters indicate significant differences among treatments at *p* < 0.05 (ANOVA and Duncan’s multiple comparison test).

**Figure 5:**
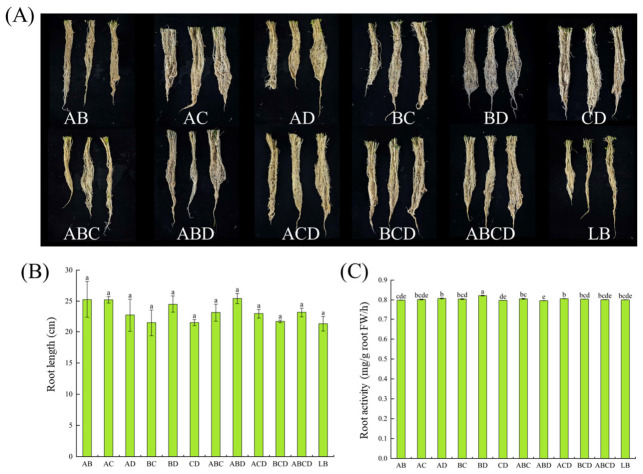
Effects of composite microbial inoculants on root length (**B**) and root vitality (**C**) of alfalfa (**A**). Values are means and bars indicate standard errors (SEs) (*n* ≥ 5). Columns with different letters indicate significant differences among treatments at *p* < 0.05 (ANOVA and Duncan’s multiple comparison test).

To evaluate the growth-promoting effects of multi-strain consortia, the previously screened efficient strains (PAP4, PA35, AC25, and TP14) were formulated into dual-strain, triple-strain, and quadruple-strain mixtures and inoculated onto alfalfa. The growth phenotypes are shown in Figure 4A. The shoot fresh weights of the BD, ABC, ABD, ACD, BCD, and ABCD treatments were significantly higher than that of the control by 13.1%, 18.2%, 11.1%, 25.3%, 14.8%, and 10.9%, respectively (*p* < 0.05) (Figure 4B); the corresponding dry weight contents also showed an increase (Figure 4C). The plant heights of the BD and ABCD treatments were significantly higher than the control by 32.0% and 14.1%, respectively (*p* < 0.05) (Figure 4D). Furthermore, the stem diameters of the BD, BCD, and ABCD treatments exceeded the control by 21.2%, 25.7%, and 22.8%, respectively (*p* < 0.05) (Figure 4E). Measurements of chlorophyll content revealed that chlorophyll a and b levels in the AB, AC, CD, ABC, and ABCD treatments were significantly higher than those of the control, while no significant differences were observed in carotenoid content (Figure 4F,G). The root lengths of the AB, AC, BD, ABC, ABD, and BCD treatments were 20.8%, 20.1%, 10%, 21.0%, and 10.1% higher than the control, respectively (*p* < 0.05) (Figure 5B). No significant differences were found in root activity among the various microbial consortia treatments (Figure 5C).

### 3.4. Effects of PGPR Inoculant on Total Nitrogen, Total Phosphorus and Crude Ash of Alfalfa

**Figure 6:**
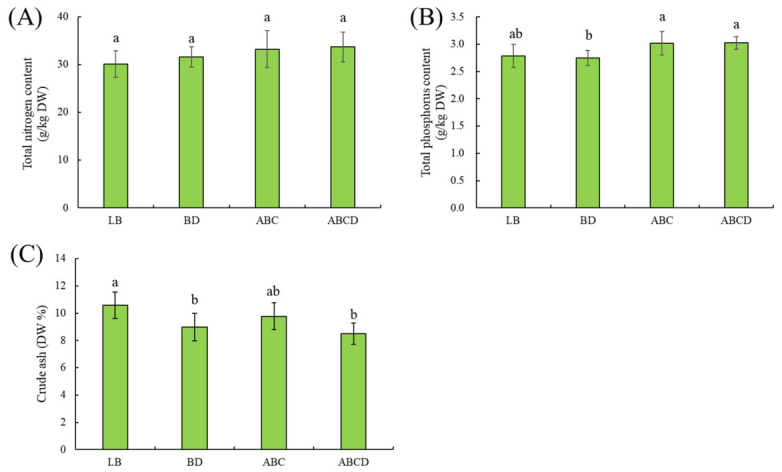
Effects of composite microbial inoculants BD, ABC, and ABCD on total nitrogen (**A**), total phosphorus (**B**), and crude ash content (**C**) of alfalfa. Values are means and bars indicate standard errors (SEs) (*n* = 3). Columns with different letters indicate significant differences among treatments at *p* < 0.05 (ANOVA and Duncan’s multiple comparison test).

Based on the aforementioned results, we further measured the concentrations of total nitrogen, total phosphorus, and crude ash in the shoots of Medicago sativa under the inoculant combinations BD, ABC, and ABCD. The results are shown in Figure 6. No significant differences were observed in TN content across treatments. However, the TN content in the three-strain combination (ABC) and the four-strain combination (ABCD) was 10.2% and 11.8% higher than the control, respectively (*p* < 0.05) (Figure 6A). Under the ABC and ABCD treatments, the TP content exceeded the control by 8.6% (*p* < 0.05) (Figure 6B). The crude ash content in all inoculant combination treatments was lower than that of the control. Specifically, the BD and ABCD treatments were 17.9% and 24.5% lower than the control, respectively (*p* < 0.05) (Figure 6C).

## 4. Discussion

The plant rhizosphere is a specialized soil niche profoundly influenced by root activity, serving as the most dynamic zone for interactions between plant metabolism and soil microorganisms [30,31,32]. Through long-term co-evolution, plants and rhizosphere microbes in diverse habitats have developed specific mutualistic symbiotic patterns [33]. However, most microbial inoculants currently used in agriculture are non-indigenous strains. Upon introduction into the soil, these strains face intense competition with native microbial communities, and their competitive fitness directly determines their practical efficacy [2]. Consequently, enhancing the competitive survival of PGPR within target habitats has become a critical challenge in the current research and development of microbial inoculants. To address this challenge, scientists have primarily focused on two dimensions to enhance the competitiveness of microbial inoculants: first, strain bioprospecting based on the principle of “habitat adaptation”. This involves screening functional strains from various stress environments, for example, isolating salt-tolerant strains from saline–alkali land to help crops alleviate salt stress, or selecting strains with high nutrient utilization efficiency from nutrient-poor soils. This approach leverages the innate adaptability of these strains to ensure a competitive survival advantage in similar target habitats [34]. Second, the construction of multi-species microbial consortia. By scientifically formulating strains with diverse functions (such as *Bacillus* and *Pseudomonas*), researchers can foster functional complementarity and synergistic effects. This leads to the formation of stable microbial communities that drive the rhizosphere micro-ecology toward a direction beneficial for plant growth [35,36].

Based on the current research status described above, this study isolated and characterized nine PGPR strains with diverse growth-promoting functions from the rhizosphere soil of *Phragmites australis*, *Triglochin maritima*, and *Alhagi maurorum* in the arid regions of Western China. Qualitative and quantitative assays for nitrogen fixation, phosphate solubilization, ACC deaminase activity, siderophore production, and the secretion of phytohormones and exopolysaccharides (EPS) revealed that all nine strains possess typical PGPR traits. Notably, most strains exhibited nitrogen fixation capacity, the ability to secrete zeatin, EPS, and siderophores, and robust salt tolerance (Table 4; Figure 1A). Furthermore, the VOCs produced by these strains significantly promoted the growth and root development of Arabidopsis thaliana (Figure 2). Building on these findings, and following PGPR inoculant design principles, four representative strains (PAP4, PA35, AC25, and TP14) belonging to different genera and possessing distinct growth-promoting strengths, were selected for pot experiments. These strains were applied to alfalfa as individual inoculants and in combinations of two, three, and four strains. While these treatments significantly enhanced alfalfa growth (Figure 3, Figure 4 and Figure 5), we observed that the growth-promoting efficacy of certain consortia was lower than that of individual strain treatments. This further demonstrates that the formulation of microbial consortia is not a simple additive effect, and the synergistic interactions between strains require deeper investigation. In conclusion, the nine PGPR strains identified in this study represent promising PGPR resources for future studies on microbial inoculant development. However, further biosafety evaluation and field validation are required before practical application.

Biological Nitrogen Fixation (BNF) is the primary pathway for nitrogen accumulation in nature. Inoculation with nitrogen-fixing PGPR can enhance plant yields, assist in disease management, and promote overall growth [37]. Most of the PGPR strains screened in this study possess nitrogen-fixation capacity (Table 4). Phosphate-solubilizing microorganisms (PSMs) lower the environmental pH by secreting organic acids, thereby dissolving phosphorus for plant uptake [22]. Strain TP8 screened in this study exhibited strong phosphate-solubilizing capability, with a relatively obvious clear halo appearing around its colony. Since microorganisms in nutrient-poor environments possess a robust capacity to adapt to their habitats, both qualitative and quantitative screenings of siderophore production were conducted. The results revealed that strains PA35 and TP14 exhibit a strong capacity for siderophore secretion (Table 4, Figure 1B). Although PGPR can facilitate iron uptake for themselves and their host plants by secreting siderophores, the quantities produced often remain insufficient to fully meet plant iron requirements. Consequently, when developing microbial inoculants tailored for nutrient-poor and saline–alkali environments, the inclusion of high-secreting siderophore strains should be considered to assist plants in the uptake and utilization of deficient elements. The findings of this study demonstrate that most of the isolated strains possess PGPR traits, with each strain exhibiting a distinct profile of growth-promoting characteristics.

PGPR typically produce phytohormones, such as indole-3-acetic acid (IAA) analogs (auxins), cytokinins, or gibberellic acids, which influence plant growth [38]. For instance, inoculating wheat roots with the auxin-secreting strains *Pseudomonas extremaustralis* IB-13-1A and *Paenibacillus illinoisensis* IB 1087 significantly promoted biomass accumulation [39,40]. In the present study, the isolated strain TP12 was found to produce indole-3-acetic acid (Table 4), and inoculation with this strain also enhanced the growth of alfalfa. While the remaining strains exhibited color reactions during qualitative analysis, they were not detected during quantitative measurement. Furthermore, most strains isolated and identified in this study produced zeatin (Table 4). As a type of cytokinin, zeatin plays a vital role in promoting plant growth. Research has shown that strains *Pseudomonas putida* MTP50, *Stenotrophomonas maltophilia* MTP42, and *Pseudomonas stutzeri* MTP40, isolated from the rhizosphere of *Coleus forskohlii*, all promote plant growth through cytokinin secretion [41]. Genera such as *Arthrobacter*, *Azospirillum*, *Bradyrhizobium*, *Bacillus*, *Pseudomonas*, and *Paenibacillus* are also known to produce zeatin [42,43]. In this study, *Klebsiella*, *Bacillus*, *Serratia*, *Pseudomonas*, and *Flavobacterium* were found to secrete zeatin to varying degrees (Table 4). The complex interactions between hormones regulate plant growth [44]; thus, the growth-promoting effects observed in this study may result from the combined influence of hormones secreted by PGPR and other functional traits.

In addition to the aforementioned PGPR traits, exopolysaccharides (EPS) are defensive biological macromolecules produced by most PGPR under extreme stressors such as nutrient deficiency, salt, heat, and drought. EPS assists bacteria in attachment, environmental adaptation, and stress tolerance, serving as a critical component of microbial biofilms [45]. EPS contains various functional groups, including carboxyl, phosphate, sulfhydryl, phenolic, and hydroxyl groups, which allow it to bind various cations with differing affinities. Most bacteria isolated in this study secreted EPS to varying degrees under NaCl concentrations of 0%, 2%, 4%, 6%, and 8%. Notably, strains PA35, AC25, and TP14 were stimulated to secrete higher levels of EPS under 4%, 4%, and 2% NaCl treatments, respectively (Figure 1A). This not only aligns with their habitat characteristics but also identifies them as excellent candidate strains and sources of EPS metabolites for future microbial inoculants in saline–alkali and other abiotic stress environments, indicating their potential as PGPR candidates for further investigation.

Microbial metabolic activities release a series of complex VOCs, most of which act as semiochemicals that diffuse freely into the environment, playing a crucial role in intra- and inter-specific communication [46,47]. The influence of VOCs on plant growth depends on dose-dependent effects and the specific plant species involved, with current research primarily focusing on their impact on root development. Well-developed root systems increase surface area and improve plant nutrition, serving as a key factor by which PGPR promotes plant growth [48]. For example, volatiles from *Arthrobacter agilis* UMCV2 can promote root development in alfalfa (*Medicago sativa*) and sorghum [49]. Similarly, volatiles from strain *Bacillus siamensis* YC7012 increase the number of lateral roots as well as root hair length and density in *Arabidopsis thaliana*, while inhibiting primary root elongation [50]. In this study, plate experiments demonstrated that VOCs secreted by the isolated PGPB significantly promoted root development in Arabidopsis. This effect is likely due to the presence of auxin-like compounds within the VOCs, which synergistically regulate root architecture (Figure 2). Ultimately, a more robust root system facilitates the uptake of nutrients [51]. Changes in root system architecture (RSA) can lead to an increase in total root surface area, which is one of the primary mechanisms by which PGPR strains accelerate plant development [52]. This expansion of root surface area enhances the absorption of nutrients and water, which may explain why inoculating alfalfa with microbial consortia ultimately promoted both growth and nutrient uptake. Although extensive research has demonstrated that the secretion of phytohormones-particularly auxins-and volatile compounds are likely the main drivers of PGPR-mediated root development, the results of this study suggest that for these specific strains, the production of volatile compounds is the primary cause for the promoted root growth in Arabidopsis and alfalfa (Figure 2). However, the specific volatile compounds produced by these strains require further characterization and identification. Recent research hotspots have demonstrated that synthetic microbial communities can achieve complex functions unattainable by individual strains, thereby overcoming the functional limitations of single-strain inoculants [34]. In this study, by evaluating various combinations-including single-strain inoculation, as well as dual, triple, and quadruple-strain consortia-we found that the growth-promoting effects varied across treatments. These findings further validate how antagonistic or synergistic interactions between microbes influence plant growth-promoting outcomes. This suggests that the interplay between microbial strains must be comprehensively considered during the research and development of microbial inoculants. Ultimately, the plant growth phenotype is the integrated result of the collective performance of various PGPR functional traits. The PGPR strains screened in this study exhibit synergistic effects with alfalfa (Medicago sativa), demonstrating potential in promoting plant growth and enhancing salt tolerance.

## 5. Conclusions

In this study, nine PGPR strains were isolated and screened from the nutrient-poor regions of Western China. Through the analysis of growth-promoting traits, plate assays, and pot experiments, it was verified that these strains and their respective consortia influenced the growth of alfalfa to varying degrees. These strains represent promising candidates for future microbial inoculant development. Beyond the large-scale bioprospecting of PGPR, future research must further resolve the complex interactions between exogenous PGPR inoculants and indigenous microbial communities, as well as their resulting rhizosphere microbial interaction networks. Such insights are essential to fully harness the potential of these microorganisms in advancing sustainable agriculture.

## Figures and Tables

**Table 1:** Culture media and their components.

Medium Name	Culture Medium Components (g L^−1^)
R2A	Yeast Extract (0.5), Proteose Peptone No. 3 (0.5), Casamino Acids (0.5), Glucose (0.5), Soluble Starch (0.5), Sodium Pyruvate (0.3), K_2_HPO_4_ (0.3), MgSO_4_·7H_2_O (0.05), Agar (15 g), pH 7.2
NBRIP	Glucose (10), Ca_3_(PO_4_)_2_ (5), MgCl_2_·6H_2_O (5), MgSO_4_·7H_2_O (0.25), KCl (0.2) (NH_4_)_2_SO_4_ (0.1), Yeast Extract (0.5), Agar (15 g), pH 7.0
King’B	Proteose Peptone (20), Glycerol (10), K_2_HPO_4_·3H_2_O (1.5), MgSO_4_·7H_2_O (1.5) Agar (15 g), pH 7.0
ADF	DF, 1-Aminocyclopropane-1-Carboxylic Acid (0.3)
NFM	Malic acid (5), K_2_HPO_4_ (0.5), KH_2_PO_4_ (0.5), MgSO_4_·7H_2_O (0.2), NaCl (0.2), CaCl_2_·2H_2_O (0.02), FeSO_4_·7H_2_O (0.05), Na_2_MoO_4_·2H_2_O (0.002), Agar (15 g), pH 7.0
DF	KH_2_PO_4_ (4.0), Na_2_HPO_4_ (6.0), MgSO_4_·7H_2_O (0.2), FeSO_4_·7H_2_O (0.01), Glucose (2.0), Gluconic acid (2.0), Citric acid (2.0), (NH_4_)_2_SO_4_ (2.0), Trace Element Solution (1.0 mL/L), H_3_BO_3_ (0.3), CuSO_4_·5H_2_O (0.04), KI (0.1), FeCl_3_·6H_2_O (0.5), MnSO_4_·4H_2_O (0.4), Na_2_MoO_4_·2H_2_O (0.2), ZnSO_4_·7H_2_O (0.4)
CAS	Chrome Azurol S (0.0605), HDTMA (0.0729), FeCl_3_·6H_2_O (0.001365), Adjust to 50 mL with distilled water, Agar (15 g), pH 7.0
LB	Tryptone (10), Yeast Extract (5), NaCl (5), Agar (15 g), pH 7.0

**Table 2:** Consortia treatment labels.

Label	Components
A	PA35
B	PAP4
C	AC25
D	TP14

**Table 3:** Results of similarity comparison among isolated strains.

Strain	Similarity	Hit Taxon Name	Plants
PAP4	99.92	*Klebsiella michiganensis*	*Phragmites australis* (Cav.) Trin. ex Steud
PA35	99.93	*Bacillus wiedmannii*	*Phragmites australis* (Cav.) Trin. ex Steud
AC12	99.86	*Bacillus pumilus*	*Alhagi camelorum* Fisch.
ACP1	99.07	*Serratia plymuthica*	*Alhagi camelorum* Fisch.
AC25	99.86	*Bacillus tequilensis*	*Alhagi camelorum* Fisch.
TP7	92.48	*Bacillus arachidis*	*Triglochin palustris* L.
TP8	99.93	*Pseudomonas tensinigenes*	*Triglochin palustris* L.
TP14	99.12	*Flavobacterium sufflavum*	*Triglochin palustris* L.
TP12	83.39	*Serratia silvae*	*Triglochin palustris* L.

**Table 4:** Growth-promoting characteristics of strains.

Strains	Growth-Promoting Characteristics (Qualitative)					Secretion of Phytohormones (μg mL^−1^)	
	Nitrogen Fixation	Soluble Inorganic Phosphorus	Soluble Organophosphorus	ACC Deaminase	Siderophore	Trans-Zeatin	Indoleacetic-3-Acid
PAP4	+	−	−	+	+	12.88 f	−
PA35	−	+	−	−	+	17.17 b	−
AC12	+	−	−	+	+	23.30 a	−
ACP1	+	+	−	−	+	14.01 cd	−
AC25	+	−	−	+	−	−	−
TP7	+	+	+	−	+	17.99 b	−
TP8	+	++	−	−	−	13.49 e	−
TP12	+	−	−	−	−	−	−
TP14	+	+	−	+	++	15.27 c	14.46

Note: “−” indicates no detectable activity; “+” indicates a positive reaction; and “++” indicates a stronger positive reaction based on bacterial growth and color development on the corresponding selective medium. Different letters indicate significant differences among treatments at *p* < 0.05.

## Data Availability

The original contributions presented in this study are included in the article/Supplementary Material. Further inquiries can be directed to the corresponding author.

## Supplementary Materials

The following supporting information can be downloaded at: https://www.mdpi.com/article/10.3390/microorganisms14061275/s1**,** Figure S1: Plate figure of strain isolation and plant growth-promoting characteristic assays. (A) Plate dilution and inoculation on R2A, NFM, and NBRIP media for bacterial screening; (B) Growth performance of isolated and purified strains on phosphate solubilization medium, ACC deaminase medium, and siderophore medium.
