# Supplementary material for: Isolation, Identification, and Growth Promotion Effects of Plant Growth-Promoting Rhizobacteria on Alfalfa
Source: Microorganisms. 2026 Jun 5;14(6):1275. doi: 10.3390/microorganisms14061275 (PMC13303310; doi:10.3390/microorganisms14061275)
Supplement: Supplementary file 1 [file microorganisms-14-01275-s001.zip › microorganisms-4338635-supplementary.pdf]

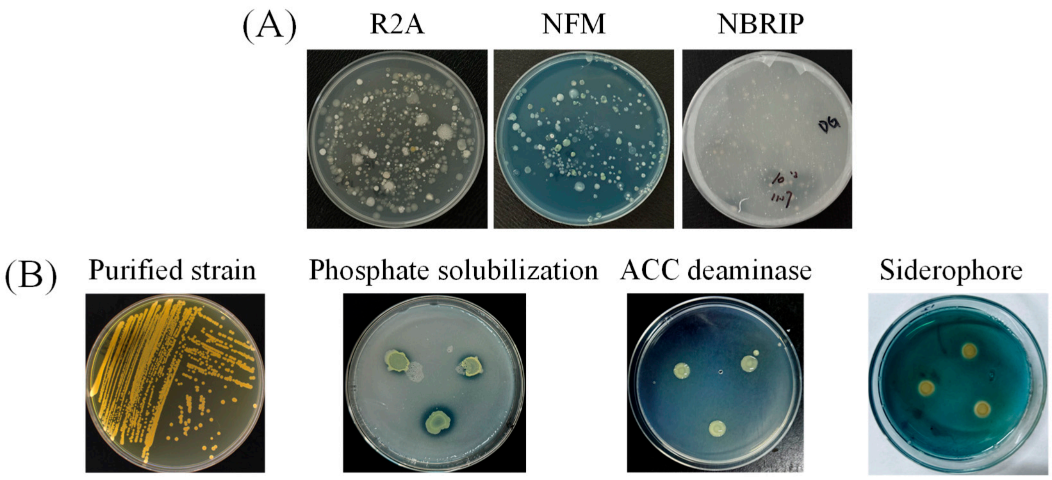

Supplementary Figure S1 Plate figure of strain isolation and plant growth-promoting characteristic assays

(A) Plate dilution and inoculation on R2A, NFM, and NBRIP media for bacterial screening; **(B)** Growth performance of isolated and purified strains on phosphate solubilization medium, ACC deaminase medium, and siderophore medium.
